# Global Gene Expression Analysis in the Livers of Rat Offspring Perinatally Exposed to Low Doses of 2,2′,4,4′-Tetrabromodiphenyl Ether

**DOI:** 10.1289/ehp.0901031

**Published:** 2009-08-17

**Authors:** Alexander Suvorov, Larissa Takser

**Affiliations:** Département Obstétrique Gynécologie, Faculté de Médecine et des Sciences de la Santé, Université de Sherbrooke, Sherbrooke, Québec, Canada

**Keywords:** genomics, low dose, metabolic pathways, microarray, PBDE, P450

## Abstract

**Background:**

Polybrominated diphenyl ethers are a group of flame-retardant chemicals appearing increasingly in the environment. Their health effects and mechanisms of toxicity are poorly understood.

**Objectives:**

We screened for the sensitive effects and mechanisms of toxicity of 2,2′,4,4′-tetrabromodiphenyl ether (BDE-47) by analyzing the gene expression profile in rats exposed to doses comparable to human exposure.

**Methods:**

Wistar dams were exposed to vehicle or BDE-47 (0.002 and 0.2 mg/kg body weight) every fifth day from gestation day 15 to postnatal day 20 by injections to caudal vein. Total RNA was extracted from the livers of pups and hybridized to the whole-genome RNA expression microarrays. The list of genes 2-fold differentially expressed was exported to PANTHER and Ingenuity Systems for analysis of enriched ontology groups and molecular pathways.

**Results:**

Oxidoreductase and transferase protein families were enriched in exposed rats as were these biological process categories: carbohydrate metabolism; electron transport; and lipid, fatty acid, and steroid metabolism. Four signaling pathways (cascades of activation of drug-metabolizing enzymes) and 10 metabolic pathways were significantly enriched. Drug-metabolizing enzymes appear to be regulated by BDE-47 through an aryl hydrocarbon receptor–independent mechanism. Direct interaction with retinoid X receptor or its upstream cascade may be involved. The main metabolic effects consisted of activation of metabolic pathways: α- and ω-oxidation of fatty acids, glycolysis, and starch hydrolysis.

**Conclusions:**

Altered expression of genes involved in metabolic and signaling pathways and functions of the organism occurs after perinatal exposure of rat offspring to BDE-47 at doses relevant for the general population.

Polybrominated diphenyl ethers (PBDEs) are a group of flame-retardant chemicals added to synthetic polymers. The main concern about PBDE health effects in the general population relates to prenatal and neonatal exposure. Indeed, because of the high lipophilicity of PBDEs and their long half-life in human tissues (Geyer et al. 2004), even small daily exposure throughout life leads to substantial PBDE accumulation in tissues with high lipid content (Johnson-Restrepo et al. 2005). Animal experiments and human evidence indicate that PBDE probably mobilizes during pregnancy, increasing the exposure of the developing organism via cord blood and then via milk (Antignac et al. 2008; Schecter et al. 2006). Moreover, children are exposed postnatally to higher PBDE doses because of higher rates of dust ingestion (Wilford et al. 2005) and higher food intake per kilogram of body weight (BW) (Schecter et al. 2006).

Experiments with animal models have shown that PBDE exposure interferes with developmental processes, causing endocrine (Darnerud 2008; Zhou et al. 2001), reproductive (Kuriyama et al. 2005; Lilienthal et al. 2006; Talsness et al. 2008), and neurodevelopmental disruption (Kuriyama et al. 2005; Suvorov et al. 2008; Viberg et al. 2006). Few articles demonstrate a relationship between PBDE exposure and health outcomes in the general population (Lim et al. 2008; Main et al. 2007).

In spite of the significant number of experimental studies, the mechanisms of PBDE toxicity and the most susceptible end points remain largely unknown. The history of studies on another group of persistent and bioaccumulative organohalogens, polychlorinated biphenyls, shows that the mechanisms involved could be multiple and remain unclear despite the long period of intensive research with traditional methods (Suvorov and Takser 2008b). The rapidly developing “omics” technologies provide toxicology with high-throughput methods of screening for the molecular targets of toxicants (Henry et al. 2002) and could be used as the first step in studying emerging environmental contaminants. The development of the high-throughput screening approach corresponds to a major strategic shift in how government agencies assess chemical hazards and risks (Collins et al. 2008).

To our knowledge, full-genome gene expression microarrays have not been used to study PBDE toxicity in a developing mammal. In this study, we analyze the change in the global gene expression in liver tissue of rat offspring exposed prenatally to 2,2′,4,4′-tetrabromodiphenyl ether (BDE-47), which is the most prevalent PBDE congener found in maternal milk (Schecter et al. 2003) and cord blood (Mazdai et al. 2003).

## Material and Methods

### Animals and treatment

We obtained nine timed pregnant Wistar rats (250–300 g; Charles River Laboratories, St. Constant, Québec, Canada) on gestational day (GD) 14 and housed them in single plastic cages with a bedding of sawdust under regulated temperature (21 ± 2°C), relative humidity (50 ± 10%), and a 12-hr light/dark cycle. Food (Charles River Rodent chow 5075) and water were provided *ad libitum*. All animals received care in compliance with *The Guide to the Care and Use of Experimental Animals* (Canadian Council of Animal Care 1993), and the protocol was approved by our institutional animal research ethics review board.

We used the same protocol of exposure as in our previous study (Suvorov et al. 2008). In short, dams received intravenous caudal injections of BDE-47 (Chromatographic Specialties Inc., Brockville, Ontario, Canada) at doses of 0.002 (group 2; G2) and 0.2 mg/kg BW (group 3; G3) in 0.3 ml/kg BW of vehicle: ethanol 95%, Cremophor EL, and sterile water for injections (1:1:8, v/v) from GD15 to postnatal day (PND) 20 every 5 days (a total of six injections/dam). The vehicle was administered to control dams (group 1; G1) according to the same protocol.

### RNA preparation and microarray analysis

One male and one female pup were selected randomly per litter on PND 27 and sacrificed by decapitation. Whole livers were harvested from these pups, snap-frozen in liquid nitrogen, and stored at −80°C until RNA extraction. RNA was extracted with the RNeasy Mini Kit (Qiagen, Valencia, CA, USA) according to the manufacturer’s instructions.

RNA samples were processed for hybridization by the Functional Genomics Platform, a collaboration between McGill University and Genome Quebec Innovation Centre (McGill University, Montreal, Canada). RNA and cRNA quality control was performed with the Agilent (Agilent, Santa Clara, CA, USA) bioanalyzer. cRNA was prepared using the Illumina RNA amplification kit (Ambion, Austin, TX, USA). cRNA was biotynilated during the *in vitro* transcription reaction. cRNA was then hybridized to the whole-genome RNA expression BeadChips RatRef-12 (Illumina Inc., San Diego, CA, USA) containing 22,523 50-mer probes. The chips were scanned with the Illumina BeadArray Reader.

### Data analysis

Data were analyzed using FlexArray 1.2 software (Blazejczyk et al. 2007). The row data were preprocessed and normalized with the lumi Bioconductor package (Du et al. 2007).

The expression values in log2 scale were analyzed with ANOVA, followed by a two-sample Bayesian *t*-test (Fox and Dimmic 2006) to identify differentially expressed genes. The false discovery rate (FDR) approach (Benjamini and Hochberg 1995) was used to correct for multiple testing. The list of genes 2-fold over- or underexpressed significantly (*p* ≤ 0.05; FDR corrected) was generated.

A two-sample Bayesian *t*-test with the FDR correction was run first to compare gene expression in male and female offspring. No differentially expressed genes (*p* ≤ 0.05) were observed; female and male data were pooled within exposure groups for subsequent analysis.

### qRT-PCR

Quantitative real-time polymerization chain reaction (qRT-PCR) was performed to quantify gene expression to validate the gene expression data obtained from microarray analysis (Table 1). Total RNA were reverse transcribed using Oligo(dT)15 Primer (Promega, Madison, WI, USA) in the presence of Omniscript Reverse Transcriptase according to the Qiagen protocol (Omniscript RT kit; Qiagen). qRT-PCR was carried out with the Eppendorf RealPlex System (Eppendorf, Hamburg, Germany) according to the protocol for the TAKARA SYBR Green kit. All samples were run in triplicate reactions. Relative gene expression was analyzed according to the 2^−ΔΔCt^ method (Livak and Schmittgen 2001). The housekeeping gene phosphofructokinase was used for normalization of the expression data. All the primers were designed using Primer-BLAST software (http://www.ncbi.nlm.nih.gov/tools/primer-blast/) and the Refseq RNA database [National Center for Biotechnology Information (NCBI) 2009a], and purchased from Integrated DNA Technologies, Inc. (Coralville, IA, USA).

### Gene ontology and pathway analysis

The lists of genes expressed differently in response to BDE-47 exposure were imported into PANTHER (http://www.pantherdb.org/), and the number of genes in each functional classification category was compared against the number of genes from NCBI’s *Rattus norvegicus* genome (NCBI 2009b) in that category. The binomial test was used to statistically determine overrepresentation of PANTHER classification categories. Bonferroni-corrected *p*-values < 0.05 were considered significant.

The same sets of genes were also imported into Ingenuity Pathway Analysis (IPA) (Ingenuity Systems Inc., Redwood City, CA, USA). Canonical pathway analysis identified canonical pathways from the IPA library that were most significant to the data set. The significance of the association between the data set and the canonical pathway was measured in two ways. First, a ratio of the number of genes from the data set that map to the pathway divided by the total number of genes that map to the canonical pathway was determined. Second, Fischer’s exact test, followed by Benjamini–Hochberg (BH) multiple testing correction, was used to calculate a *p*-value determining the probability that the association between the genes in the data set and the canonical pathway can be accounted for by chance only.

### Blood cholesterol and triglycerides analysis

Cholesterol and triglycerides were determined in trunk blood of pups sacrificed on PND 27. Analysis was performed at the Centre Hospitalier Universitaire de Sherbrooke (CHUS) Clinical Laboratory by Vitros 950 Chemistry System (Ortho-Clinical Diagnostics, Rochester, NY, USA).

## Results

We found no significant relationship between litter size and dose of exposure to BDE-47, with the number of pups varying from 10 to 17 per litter. No weight differences were observed between the control and exposed dams throughout the experiment. The BW of pups from both exposed groups increased; this finding is described in detail elsewhere (Suvorov et al. 2009).

### Gene expression profile

**Figure 1:**
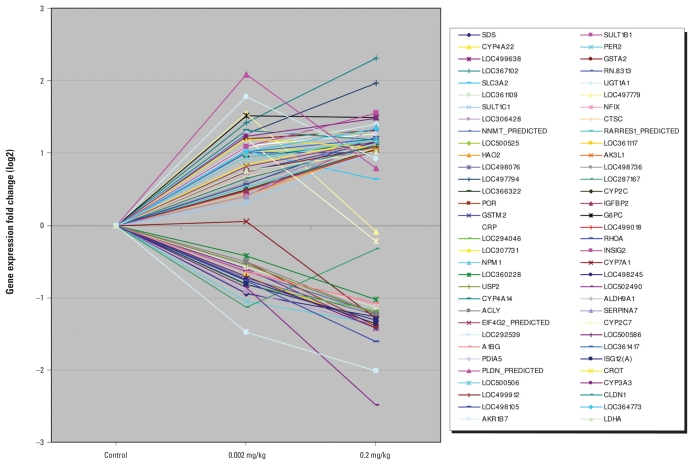
Expression of 66 genes in the livers of the control group and two groups of rats perinatally exposed to 0.2 and 0.002 mg/kg BW of BDE-47. The genes illustrated were 2-fold differentially expressed (*p* ≤ 0.05; FDR corrected) in either exposed group.

Sixty genes were 2-fold differentially expressed (*p* ≤ 0.05; FDR corrected) in livers of the G3 rats [see Supplemental Material, Table 1, available online (doi:10.1289/ehp.0901031.S1 via http://dx.doi.org/)]: 22 were underexpressed and 38 were overexpressed. Twenty-one genes were 2-fold differentially expressed (*p* ≤ 0.05; FDR corrected) in G2: 3 were underexpressed and 18 were overexpressed. Monotonic dose–response relationships were observed for most of the genes 2-fold differentially expressed in both groups (Figure 1). The response of 6 genes appears to be nonlinear: 1 gene (*LOC287167*) was underexpressed, and 5 (*SLC3A2, LOC497779, CYP2C7, LOC292539, PLDN_PREDICTED*) were overexpressed in G2, whereas their expression in G3 was not significantly different from the control. Expression of 12 genes selected randomly was validated by qRT-PCR. A high correlation was observed between PCR and microarray data (Table 1).

### PANTHER gene ontology analysis

Among molecular functions, the oxidoreductase protein family with oxygenase subfamily was enriched (*p* ≤ 0.05, Bonferroni corrected) in the livers of both groups of exposed rats (Table 2). The transferase family was significantly overrepresented in G3, and a similar trend was characteristic for G2.

These PANTHER biological process categories were enriched in both exposed groups (Table 2): carbohydrate metabolism; electron transport; and lipid, fatty acid, and steroid metabolism. Within the limits of the last category, fatty acid and steroid metabolism were significantly overrepresented. Steroid-hormone metabolism was overrepresented within the limits of steroid metabolism category. The enrichment of carbohydrate metabolism and steroid-hormone metabolism in G2 was not significant but followed the same trend.

### Ingenuity Pathway Analysis

We then used IPA to perform pathway analysis of the differentially expressed gene sets. Of the data set of 66 differentially expressed genes, 59 were mapped in Ingenuity Knowledge Base; 27 genes populated the functions/pathways category.

**Figure 2:**
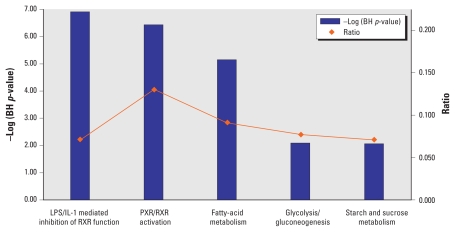
IPA generated canonical pathways enriched in the liver of rats exposed to BDE-47. Ratio, number of 2-fold differentially expressed genes that map to a pathway divided by the total number of genes in Illumina RatRef-12 annotation that map to the canonical pathway; *p*-value, probability that the association between the 2-fold differentially expressed genes and the canonical pathway can be accounted for by chance only.

Fourteen canonical pathways were significantly enriched in the liver samples obtained from G2 and G3 (Table 3): 4 signaling pathways dealing mainly with activation of drug metabolizing enzyme cascade; 10 metabolic pathways including 4 pathways of lipid metabolism, 2 pathways of amino acid metabolism, 2 pathways of carbohydrate metabolism, 1 pathway of cofactor and vitamin metabolism, and 1 pathway of complex carbohydrate metabolism. Number of pathways were enriched because the same differentially expressed genes populated multiple pathways. The optimized set of pathways obtained after exclusion of most of overlapping pathways is shown in Figure 2 [see also Supplemental Material, Figure 1 (doi:10.1289/ehp.0901031.S1)].

### Blood cholesterol and triglycerides

**Figure 3:**
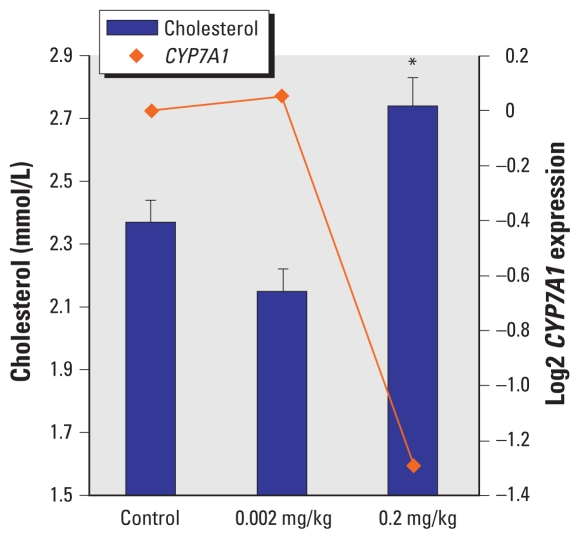
Blood level of cholesterol (mean ± SE) and *CYP7A1* expression in rat offspring exposed to low doses of BDE-47 on PND 27. *Dunnett’s *p* = 0.007

No significant difference was observed in triglycerides blood level in control and exposed pups on PND 27 (data not shown). Blood level of total cholesterol in G3 (adjusted for litter size, individual weight, and sex) was significantly higher than in G1. No significant differences were observed between G1 and G2. Blood cholesterol coincided with underexpression of CYP7A1 in the same rats (Figure 3).

## Discussion

To our knowledge, this is the first study of PBDE effects on a mammal using high-throughput genomic methods. We obtained evidence of prolonged effects of perinatal exposure to low doses of BDE-47 on gene expression profile in the liver tissues of rat offspring. Significant 2-fold changes in gene expression were observed in pups 1 week after the last indirect (via milk) administration of BDE-47. The observed change in gene expression was dose dependent for the majority of altered genes. Multiple signaling and metabolic pathways were affected by BDE-47 or its metabolites in our study.

The protocol of exposure used in this study was designed to simulate exposure of the general population. In contrast to most published experimental studies of PBDE toxicity, we used an intravenous route of exposure for dams to assure the accuracy of internal dosing. As we used very low doses (the high dose used herein, 0.2 mg/kg BW, is among the lowest ever used in experimental studies), intravenous administration allowed avoiding uncertainty in the amount of BDE-47 received. In fact, the rat offspring studied in our experiments received BDE-47 via cord blood and via milk. As BDE-47 was previously detected in both human cord blood and milk (Frederiksen et al. 2009), the chosen route of administration has relevance for humans. The choice of doses in our study was verified by internal doses estimated earlier (Suvorov et al. 2008): BDE-47 concentrations in subcutaneous fat of the dams and pups 1 week after the last injection corresponds to the levels observed in the North American human population.

One of the major findings of the current study is the differential expression of phase I and II metabolic enzymes. This finding is supported by the enriched PANTHER molecular function categories (oxidoreductase and transferase) and biological process categories (electron transport) as well as the following significantly enriched canonical pathways according to IPA analysis: LPS/IL-1–mediated inhibition of retinoid X receptor (RXR) function; pregnane X receptor (PXR)/RXR activation; xenobiotic metabolism signaling; and metabolism of xenobiotics by cytochrome P450 (CYP). All these pathways overlap and partly describe the same cellular processes [see Supplementary Material (doi:10.1289/ehp.0901031.S1)].

Because induction of cytochrome enzymes may alter numerous metabolic pathways and lead to the formation of oxygen radicals and/or carcinogens, the study of hepatic-enzyme induction has remained within the focus of experimental toxicology from the earliest PBDE studies. It has been demonstrated that both the commercial mixtures and BDE-47 congener induced phase I EROD (ethoxyresorufin-*O*-deethylase) and PROD (pentoxyresorufin-*O*-deethylase) and phase II metabolic enzyme activity (uridinediphosphate-glucuronosyltransferase) (Carlson 1980; Hallgren and Darnerud 1998; von Meyerinck et al. 1990; Zhou et al. 2001). In all these studies, however, the named induction was observed at levels of exposure exceeding many-fold environmental exposures (ranging usually from tens to hundreds of milligrams per kilogram BW).

In a study by Sanders et al. (2005), liver expression of *CYP1A1, CYP2B,* and *CYP3A* were analyzed after oral administration of DE-71 as well as individual congeners to adult rats on 3 consecutive days in doses ranging from 1.5 to 150 μmol/kg BW/day. The cytochromes chosen were biomarkers for activation of aryl hydrocarbon receptor (AhR), constitutive androstane receptor (CAR), and PXR. The authors concluded that PBDEs, being non-coplanar organohalogens, are very poor AhR inducers and that most of previously reported AhR-inducing properties of PBDE mixtures can be explained by the presence of polybrominated dibenzofurans (PBDF) and polybrominated dibenzodioxins (PBDD) in the mixtures. However, the significant up-regulation of *CYP2B* and *CYP3A* by BDE-47 was also achieved at high doses of 4.9 and 49 mg/kg BW/day, respectively.

Similar conclusions concerning the role of PBDD and PBDF in activating AhR by low-grade purified BDE-47 were reported by Wahl et al. (2008) based on a study of AhR-mediated toxicity and gene expression in rat hepatoma cells and in zebrafish embryos. In addition, *CYP2B* and *CYP3A* were found to be overexpressed in this study.

Pacyniak et al. (2007) found that *CYP3A11* and *CYP2B10* (but not *CYP1A1*) were overexpressed in adult mice livers after 4 consecutive days of intraperitoneal injections of 10 and 100 μmol of different congeners of PBDE (4.9 and 49 mg/kg/BW/day for BDE-47). Because the two induced cytochromes are known target genes of PXR, the authors conclude that PBDE congeners are PXR activators. This conclusion was supported by luciferase assays, which showed that BDE-47, 99, and 209 activated PXR and its human counterpart, steroid X receptor, but not AhR.

Hence, the evidence obtained by several groups (Pacyniak et al. 2007; Sanders et al. 2005; Wahl et al. 2008) supports the idea that PBDE induces CYP by a mechanism other than AhR mediation. At the same time, induction of CYPs that are targets for receptors other than AhR receptors were also observed at the high doses of PBDE exposure.

According to our data, no CYPs were induced in the AhR pathway. Moreover, all three molecules, GSTM1, GSTA5 and ALDH9A1, differentially expressed downstream of AhR are also regulated through the CAR. It could be assumed, therefore, that AhR does not participate at all in PBDE-induced response.

Six CYPs (*CYP2C, CYP2C7, CYP3A23/3a1, CYP4A1, CYP4A3,* and *CYP7A1*) were 2-fold differentially expressed in our study (Table 3). According to current knowledge, these cytochromes are targets of several nuclear receptors. CAR may participate in the induction of *CYP2C* (Gerbal-Chaloin et al. 2002), *CYP2C7* (Chen et al. 2003), and *CYP3A23* (Sueyoshi and Negishi 2001). These three CYPs can be induced by PXR (Kliewer et al. 2002). *CYP4A3* and *CYP4A1* are known to be regulated by peroxisome proliferator-activated receptor alpha (PPARα) (Kroetz et al. 1998). Finally, multiple mechanisms are involved in the control of *CYP7A1* transcription, and a variety of transcription factors and nuclear receptors participate in the sophisticated regulatory networks (Gilardi et al. 2007), including PXR, liver X receptor alpha (LXRα), farnesoid X receptor, and CAR. The common feature of all these receptors is that they represent the dimerization partners for RXRs. In context of RXR pathway, a number of genes were significantly overexpressed at the smallest ever studied exposure doses of BDE-47, 0.002 mg/kg BW. The direct interaction of PBDE with RXR or upstream mechanisms of RXR induction cannot be excluded in light of this evidence. The possibility of direct interaction of xenobiotics with RXRs was recently demonstrated (Alsop et al. 2003; Li et al. 2008) as well as possibility of induction of PPAR and LXR pathways by RXR-selective ligands (le Maire et al. 2009; Mukherjee et al. 1997).

Regulation of several CYPs consistent with activation of PXR may affect concentrations of molecules important for the regulation of the physiological functions of an organism. Thus, according to the enriched PANTHER biological processes, steroid-hormone metabolism was altered in the rat offspring exposed to BDE-47. Two genes activated by PXR, *CYP3A23* and *UGT1A1*, are involved in testosterone hydroxylation and glucuronidation, respectively. These reactions result in products that are more polar, water soluble, and more easily excreted from the body than the parent compound (Bélanger et al. 2003). Increased testosterone excretion may be responsible for the impairment of sexual development observed in the PBDE-exposed rats, namely, decreased sperm count, feminization of sexually dimorphic behavior, and decreased anogenital distance in rat male offspring (Kuriyama et al. 2005; Lilienthal et al. 2006). The anogenital distance was significantly decreased on PND 66 in the male offspring exposed to 0.2 mg/kg BW BDE-47 from the same litters studied herein (Suvorov and Takser 2008a).

Activation of PXR may be responsible as well for down-regulation of CYP7A1, the rate-limiting enzyme of bile-acid biosynthesis from cholesterol, in G3. In G2, the same enzyme was slightly overexpressed. This nature of nonlinear dose–response expression of *CYP7A1* is unclear and probably relates to the sophisticated network of receptors and transcription factors involved in its regulation (Gilardi et al. 2007).

The next major finding consists of activation of a number of metabolic pathways, including metabolism of lipids, carbohydrates, and amino acids. In fact, up-regulation of glucose-6-phosphate phosphatase (G6PC) causes uptake of glucose and its retention in cytoplasm. Overexpression of *G6PC* corresponds to the previously reported (Suvorov et al. 2009) observation of increased recovery rate of blood glucose levels after oral glucose administration in exposed rats compared with controls. SLC3A2, responsible for the starch hydrolysis with dextrin production, which hydrolyzes further into glucose, is overexpressed as well. Further, *LOC500506* is upregulated. This gene is similar to another key enzyme, glyceraldehyde-3-phosphate dehydrogenase (GAPDH), which catalyzes the first reaction of the “payoff” phase of glycolysis. Two enzymes responsible for lactate transformation into acetyl-CoA are up-regulated as well. Finally, alternative α and ω pathways of oxidation of fatty acids are activated in the exposed groups of rats (Table 3). Alteration of carbohydrate metabolism genes, along with genes involved in proton transport, was shown recently in zebrafish embryonic fibroblasts after the treatment by 6-hydroxy-2,2′,4,4′-BDE-47 (van Boxtel et al. 2008). Activation of metabolic pathways corresponds to our previous finding (Suvurov et al. 2009) of increased growth and elevated blood IGF-1 in rat offspring exposed to low doses BDE-47, using the same protocol as in the present study. In fact, IGF-1 and somatotropin are known to be powerful anabolism promoters (Scarth 2006). In this regard, it is interesting to mention that *IGFBP2* was significantly overexpressed by 2-fold in liver of both groups of exposed rats. IGFBP-2 demonstrates cell-surface localization, and it has been suggested that membrane-associated IGFBP-2 may concentrate the IGFs in close vicinity of their receptors, thereby increasing their mitogenic potential (Chesik et al. 2007).

## Conclusion

This study has revealed an alteration in gene expression involved in the metabolic and signaling pathways and functions of the organism after perinatal exposure of rat offspring to BDE-47 at doses relevant for general population. This analysis, based on genomic methods, yielded new information that can be used for generating a mechanistic hypothesis for further study of perinatal exposure of PDBE using traditional methods of toxicology.

## Figures and Tables

**Table 1:** Gene expression changes verified by qRT-PCR.

Gene ID	Expression fold-change (log2)				Sequence of primers used in qRT-PCR
	Illumina		qRT-PCR		
	G2^a^	G3^b^	G2^a^	G3^b^	
*AKR1B7*	−1.48	−2.02	−1.36	−1.93	TAAGCCTGAGGACCCCGTAG (F) CACGTTCCTTTGGACATGGA (R)
*POR*	−0.51	−1.39	−0.46	−1.20	ATCACCAACATGGGGGACTC (F) ACAGAACCATGTCCGTCGTG (R)
*PER2*	−1.05	−1.36	−1.21	−1.18	GCGGAAATGAAAACTGCTCC (F) CGATTCCACTAACATCCGCA (R)
*CYP7A1*	0.05	−1.29	0.13	−1.34	CGCGACAATATGTCCTGGAA (F) GGGGACACTTGACTTGGCTC (R)
*SLC3A2*	1.03	0.63	1.00	0.49	GGACACCGAAGTGGACATGA (F) CACCAGACCGTTCTTCTCCC (R)
*IGFBP2*	0.48	1.02	0.62	1.17	TTACGCTGTTACCCCAACCC (F) CACGCGTCTCTTTTCACAGG (R)
*CYP2C*	0.49	1.05	0.35	1.30	AGGAAAAGCACAATCCGCAG (F) TTTCAGCAGCAGCAGGAGTC (R)
*CLDN1*	0.65	1.11	0.55	1.25	GGCTATGAGGGTCTTGGCTG (F) TCGCCCATTTGAGTGTCATC (R)
*RHOA*	0.57	1.22	0.71	1.41	CAAGGACCAGTTCCCAGAGG (F) CCAACTCTACCTGCTTCCCG (R)
*CYP3A23*	1.22	1.48	1.10	1.68	GAAAGGCAAACCTGTCCCTG (F) GGATCCTTCGGGTTGTTGAG (R)
*G6PC*	1.52	1.50	1.65	1.63	CTACACCCTTTGCCAGCCTC (F) TTGCAGCTCTTGCGGTACAT (R)
*INSIG2*	1.09	1.55	1.01	1.48	TGGCCCCTACATTTCCTCTG (F) CAGGAACACGCCAATGAAGA (R)

Abbreviations: F, forward; R, reverse.

a Group exposed to 0.002 mg/kg BW BDE-47.

b Group exposed to 0.2 mg/kg BW BDE-47.

**Table 2:** Gene ontology analysis using PANTHER.

	0.002 mg/kg BW BDE-47		0.2 mg/kg BW BDE-47	
Molecular function	No. of genes	*p*-Value^a^	No. of genes	*p*-Value^a^
Transferase	3	9.07 × 10^−5^	9	5.86 × 10^−3^
Oxidoreductase	7	2.16 × 10^−5^	13	2.21 × 10^−7^
Oxygenase	5	1.80 × 10^−5^	6	2.65 × 10^−4^
Biological process				
Carbohydrate metabolism	3	3.00 × 10^−1^	8	1.59 × 10^−3^
Electron transport	5	1.38 × 10^−4^	7	3.23 × 10^−4^
Lipid, fatty acid, and steroid metabolism	9	1.28 × 10^−7^	12	9.37 × 10^−6^
Fatty acid metabolism	6	1.55 × 10^−6^	5	1.86 × 10^−2^
Steroid metabolism	4	4.39 × 10^−3^	7	1.76 × 10^−4^
Steroid-hormone metabolism	2	1.79 × 10^−1^	4	1.96 × 10^−3^

a Bonferroni corrected.

**Table 3:** IPA generated canonical pathways significantly enriched in liver samples obtained from rats perinatally exposed to BDE-47.

Ingenuity canonical pathways	Log (BH *p*-value)	Ratio	Genes
Signaling pathways			
LPS/IL-1 mediated inhibition of RXR function	1.22 × 10^−7^	7.10 × 10^−2^	*ALDH9A1, CYP2C, CYP2C7, CYP3A23, CYP4A1, CYP4A3, CYP7A1, GSTA5, GSTM1, SULT1B1*
PXR/RXR activation	3.60 × 10^−7^	1.30 × 10^−1^	*CYP2C, CYP2C7, CYP3A23, CYP7A1, G6PC, GSTM1, UGT1A1*
Xenobiotic metabolism signaling	2.80 × 10^−5^	4.88 × 10^−2^	*ALDH9A1, CYP2C, CYP2C7, CYP3A23, GSTA5, GSTM1, SULT1B1, UGT1A1*
AhR signaling	1.10 × 10^−2^	3.88 × 10^−2^	*GSTM1, NFIX, GSTA5, ALDH9A1*

Metabolic pathways			
Lipid metabolism			
Fatty acid metabolism	7.33 × 10^−6^	9.10 × 10^−2^	*ALDH9A1, CYP2C, CYP2C7, CYP3A23, CYP4A1, CYP4A3, SDS*
Arachidonic acid metabolism	3.96 × 10^−4^	6.94 × 10^−2^	*CYP2C, CYP2C7, CYP3A23, CYP4A11, CYP4A3*
Bile acid biosynthesis	2.86 × 10^−3^	1.15 × 10^−1^	*CYP3A23, CYP7A1, ALDH9A1*
Linoleic acid metabolism	1.42 × 10^−2^	5.56 × 10^−2^	*CYP2C, CYP3A23, CYP2C7*
Metabolism of cofactors and vitamins			
Metabolism of xenobiotics by CYP	2.80 × 10^−5^	8.10 × 10^−2^	*CYP2C, CYP2C7, CYP3A23, GSTA5, GSTM1, UGT1A1*
Carbohydrate metabolism			
Glycolysis/gluconeogenesis	8.29 × 10^−3^	7.70 × 10^−2^	*G6PC, LDHA, ALDH9A1*
Propanoate metabolism	8.68 × 10^2^	7.32 × 10^−2^	*SDS, LDHA, ALDH9A1*
Amino acid metabolism			
Tryptophan metabolism	8.60 × 10^−4^	5.88 × 10^−2^	*ALDH9A1, CYP2C, CYP2C7, CYP3A23, SDS*
Cysteine metabolism	1.89 × 10^−3^	1.30 × 10^−1^	*SDS, SULT1B1, LDHA*
Metabolism of complex carbohydrates			
Starch and sucrose metabolism	8.68 × 10^−3^	7.14 × 10^−2^	*G6PC, UGT1A1, SLC3A2*
